# In vitro and in vivo uptake studies of PAMAM G4.5 dendrimers in breast cancer

**DOI:** 10.1186/s12951-016-0197-6

**Published:** 2016-06-13

**Authors:** Natalia Oddone, Nicole Lecot, Marcelo Fernández, Alejandra Rodriguez-Haralambides, Pablo Cabral, Hugo Cerecetto, Juan Claudio Benech

**Affiliations:** Laboratorio de Señalización Celular y Nanobiología, Instituto de Investigaciones Biológicas Clemente Estable, Av. Italia 3318, CP 11600 Montevideo, Uruguay; Centro Interdisciplinario de Nanotecnología, Química y Física de Materiales, Espacio Interdisciplinario, UdelaR, José E. Rodó 1843, CP 11200 Montevideo, Uruguay; Área de Radiofarmacia, Centro de Investigaciones Nucleares, UdelaR, Mataojo 2055, CP 11400 Montevideo, Uruguay; Laboratorio de Experimentación Animal, Centro de Investigaciones Nucleares, UdelaR, Mataojo 2055, CP 11400 Montevideo, Uruguay; Química Bioanalítica, Instituto Polo Tecnológico de Pando, Facultad de Química, UdelaR, By Pass de Pando y Ruta 8, CP 91000 Pando, Canelones Uruguay

**Keywords:** PAMAM G4.5 dendrimers, Antitumor drug delivery systems, Breast cancer treatment or diagnosis

## Abstract

**Background:**

Breast cancer is the second leading cause of cancer death worldwide. Nanotechnology approaches can overcome the side effects of chemotherapy as well as improve the efficacy of drugs. Dendrimers are nanometric size polymers which are suitable as drug delivery systems. To the best of our knowledge, studies on the application of PAMAM G4.5 (polyamidoamine half generation 4) dendrimers as potential drug delivery systems in breast cancer have not been reported. In this work we developed a PAMAM G4.5 dendrimer containing FITC (fluorescein isothiocyanate) dye to study their uptake by murine breast cancer cells and BALB/c mice breast tumors.

**Results:**

We performed a reaction between FITC and PAMAM G4.5 dendrimers which were previously derivatized with piperazine (linker molecule), characterized them by ^1^H NMR (proton nuclear magnetic resonance) spectroscopy and MALDI-TOF (matrix-assisted laser desorption/ionization- time-of-flight) mass spectrometry. The experimental data indicated that 2 FITC molecules could be bound covalently at the PAMAM G4.5 dendrimer surface, with 17 FITC molecules probably occluded in PAMAM dendrimers cavity. PAMAM-FITC dendrimer (PAMAM G4.5-piperazinyl-FITC dendrimer) size distribution was evaluated by DLS (dynamic light scattering) and TEM (transmission electron microscopy). The nanoparticle hydrodynamic size was 96.3 ± 1.4 nm with a PdI (polydispersion index) of 0.0296 ± 0.0171, and the size distribution measured by TEM was 44.2 ± 9.2 nm. PAMAM-FITC dendrimers were neither cytotoxic in 4T1 cells nor hemolytic up to 24 h of incubation. In addition, they were uptaken in vitro by 4T1 cells and in vivo by BALB/c mice breast tumors. PAMAM G4.5-piperazinyl-FITC dendrimer intracellular distribution was observed through histologic analysis of the tumor by laser confocal microscopy.

**Conclusion:**

These results indicate that PAMAM G4.5 dendrimers enter tumor tissue cells, being good candidates to be used as antitumor drug delivery systems for breast cancer treatment and diagnosis.

## Background

Breast cancer remains the most common invasive cancer in women and is the second leading cause of cancer death worldwide [1–4]. Chemotherapy of solid tumors, including breast cancer, is linked to several side effects [5]. Nanotechnology provides the possibility of creating delivery systems that reduce the unwanted side effects of systemic delivery, increasing tumor accumulation and improving efficacy [6]. The EPR effect (enhanced permeability and retention effect) is responsible for the aforementioned advantages. The EPR effect arises due to differences between the blood vessels surrounding the tumors and those of normal tissues [7]. Blood vessels in tumors are defective and the endothelial cells in the vessels are poorly aligned [8]. Owing to the permeability of tumor blood vessels, nanosized vehicles would pass through into the tumor but not into healthy tissues [7]. The EPR effect also allows nanovehicles to accumulate in the tumor site due to poor lymphatic drainage [4, 8, 9]. Therefore, EPR effect is an important pharmacokinetic principle to take into account in the design of drug delivery nanosystems [10]. Since its description by Matsumura and Maeda in 1986, several experimental evidences have confirmed the existence of this effect in solid tumors [11–14].

PAMAM dendrimers are branched macromolecules with multiple end-groups that can be functionalized with different molecules, including drugs [15]. Drugs can also be encapsulated into dendrimer cavities which makes them suitable as drug delivery systems [16, 17].

In order to use dendrimers as drug delivery systems for cancer therapy, it is important to know if they are uptaken by the tumor. There are several studies reporting the uptake of full generation dendrimers (cationic dendrimers) in different cell lines [18–21]. This could be attributed to the fact that full generation dendrimer labeling is simpler than half generation dendrimer (anionic) labeling. However, recent evidence underlines the influence of charge in dendrimer cytotoxicity, being full generation dendrimers more toxic than half generation dendrimers [22–24]. This toxicity is related to dendrimers surface charge; positively charged dendrimers are more toxic than negative charged dendrimers [25–27]. Full generation dendrimers become biocompatible when functionalized with certain molecules because of neutralization of positive charges [28].

There are few reports on half generation PAMAM dendrimers in cancer applications [8, 29]. For instance, Kirkpatrick et al. [8] in 2011, evaluated anionic half generation (G3.5–G6.5) PAMAM dendrimers as delivery vehicles of cisplatin for ovarian cancer treatment [8] and Morgan et al. in 2006, employed a polyester G4.5 dendrimer composed of glycerol and succinic acid to deliver antitumor camptothecins in cancer cell lines [30]. On the other hand, Kitchens et al. studied the uptake of PAMAM dendrimers of half generation (G1.5–G3.5) in Caco-2 cells and they conjugated the dendrimers with FITC to this end. These researchers employed an ethylenediamine moiety as linker group and did not describe spectroscopic characterization of the prepared fluorescent dendrimers [31]. A recent work from Yuan et al. 2011, has conjugated PAMAM G4.5 dendrimers with AAF (5-(aminoacetamido)fluorescein), to track transbuccal transport of these dendrimers as potential central nervous system therapeutic nanoparticles [32]. They compared the permeability of AAF labeled PAMAM G4.5 dendrimers with FITC labeled PAMAM G4 dendrimers. They showed that AAF-labeled PAMAM G4.5 dendrimers were less permeable than FITC-labeled PAMAM G4 dendrimers. This result was consistent with the study of Kitchens et al. [31], who showed that cationic PAMAM dendrimers can gain enhanced transport by causing opening of epithelial tight junctions and toxicity effects. These authors did not study these dendrimers as potential nanovehicles for drug delivery in breast cancer. Noteworthy, studies related to PAMAM G4.5 dendrimers cell uptake and intracellular distribution were not found in the literature, nor was the use of PAMAM G4.5 dendrimers as potential drug delivery nanosystems in breast cancer.

According to the information previously described regarding PAMAM dendrimers, dendrimer generation and dendrimer charge are physicochemical important parameters to be considered in the selection of the type of dendrimer to be used for a certain application. Thus, taking into account the absence of studies of PAMAM G4.5 dendrimers as potential drug delivery nanosystems in breast cancer and that positive charged dendrimers are more toxic than negative charged dendrimers, PAMAM G4.5 carboxylate dendrimers were selected. In the present work, we characterized the in vitro and in vivo uptake of PAMAM G4.5 carboxylate dendrimers in 4T1 tumor cells, showing for the first time that these dendrimers have a great potential as drug delivery nanosystems for breast cancer treatment and diagnosis.

## Results and discussion

### Synthesis and characterization of PAMAM G4.5-piperazinyl-FITC dendrimer

Recently, our group labeled directly PAMAM G4 dendrimers with FITC (fluorescein isothiocyanate) [15]. Fluorescein is one of the most common fluorophores employed in nanoparticle uptake studies. As the carboxylic acids groups in PAMAM G4.5 dendrimers are not able to directly bind to FITC, an adequate linker is required to this end. *N*-Monoprotected piperazine was used as the linker moiety; it is a bifunctional molecule with a free amine group and an amine group covalently bonded to a *tert*-butyloxycarbonyl protecting group (*N*-Boc). The dendrimer was first bound to the free amine group of piperazine via an amide moiety, generating PAMAM G4.5-*N*-Boc-piperazinyl dendrimer, and after *N*-Boc deprotection, it was bound to FITC as described in the “Methods” section.

**Fig. 1:**
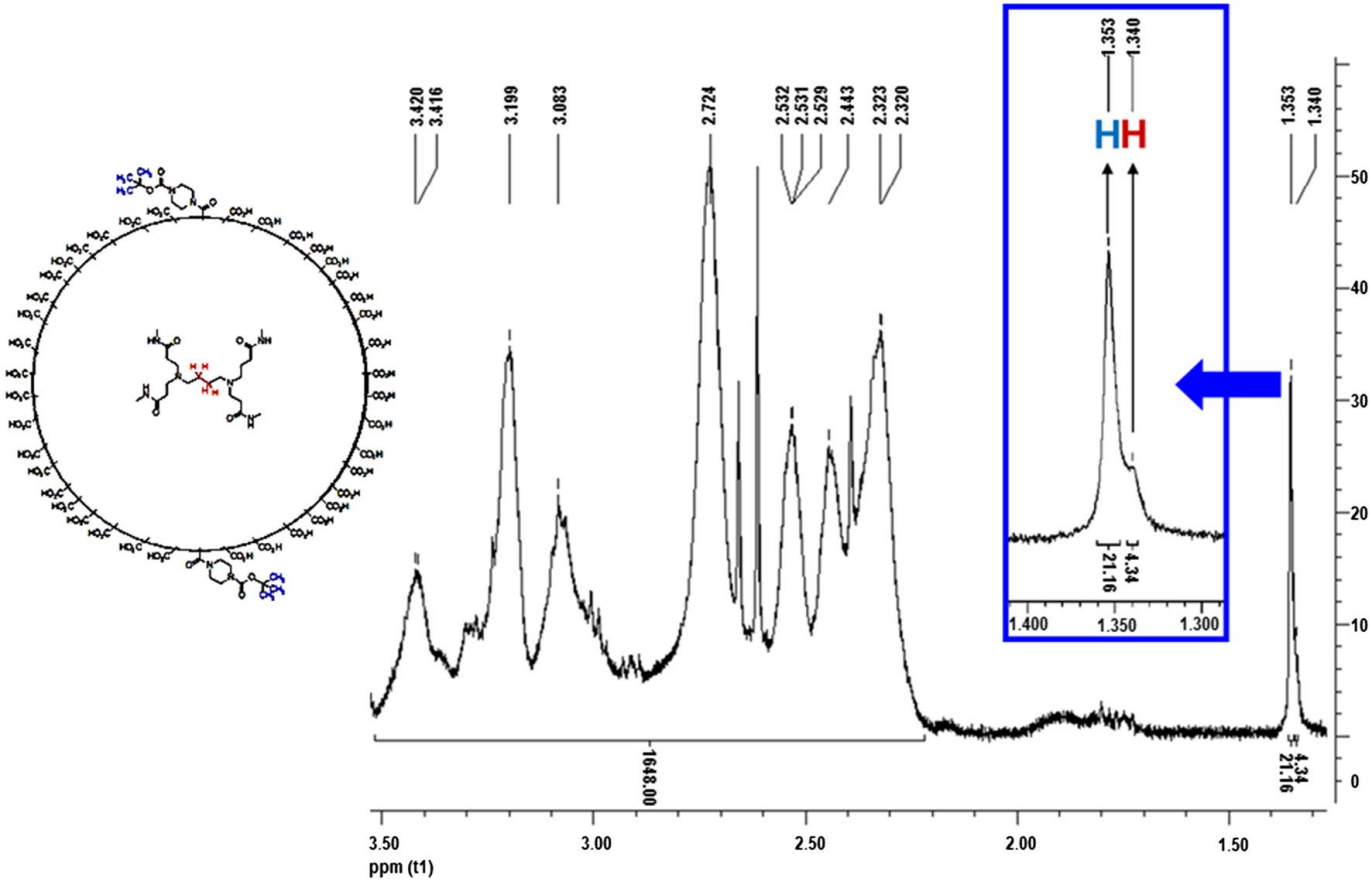
^1^H NMR of PAMAM G4.5-*N*-Boc-piperazinyl dendrimers. This spectrum was acquired in D_2_O at 303 K (number of scans 64)

The intermediate PAMAM G4.5-*N*-Boc-piperazinyl dendrimer was characterized by ^1^H NMR at 303 K in D_2_O, because this intermediate product was poorly soluble in other solvents like MeOH-*d*_4_ or DMSO-*d*_6_. The signal at 1.35 ppm (**H**, Fig. 1) in ^1^H NMR spectrum that corresponds to the protons of *t*-butyl methyl groups of *N*-Boc-piperazine, confirms the formation of the amide. Besides, the signal at 1.34 ppm (**H**, Fig. 1) corresponds to the protons of methylene groups at the core of PAMAM G4.5 dendrimers (Fig. 1). According to the integration of the signals of ^1^H NMR spectra, it can be confirmed that piperazine was covalently bonded to PAMAM G4.5 dendrimers in an *N*-Boc-piperazine: PAMAM G4.5 dendrimer ratio of 2:1 (Fig. 1).

After *N*-Boc group elimination, using trifluoroacetic acid, the subsequent reaction of the piperazinyl-dendrimer derivative with FITC was performed. This synthetic procedure was performed five times. The PAMAM-FITC dendrimers were characterized, by ^1^H NMR at 303 K in D_2_O, owing to the final product was poorly soluble in other solvents like MeOH-*d*_4_ or DMSO-*d*_6_.

**Fig. 2:**
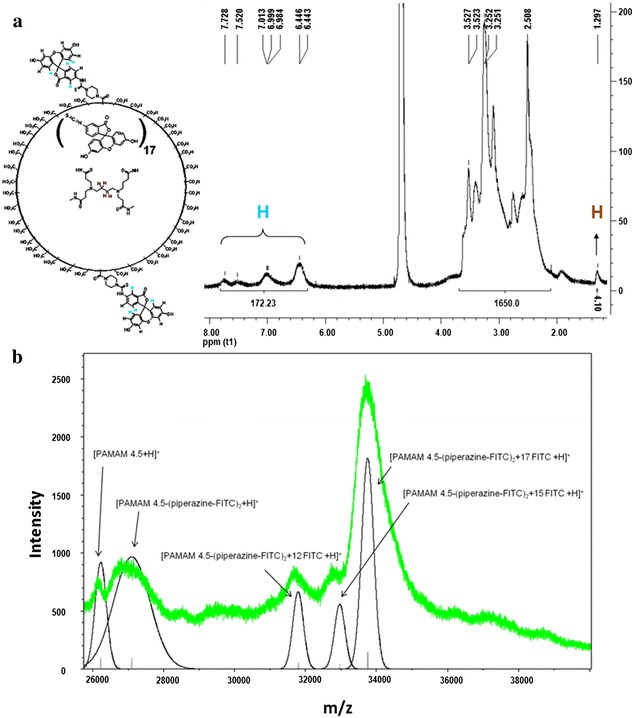
Structural characterization of PAMAM-FITC dendrimers. **a** ^1^H NMR spectrum was acquired in D_2_O at 303 K (number of scans 64). **b** MALDI-TOF mass spectrometry. Experimental data (*green trace*) and calculated spectra (*black trace*)

In all the cases (n = 5) the ^1^H NMR spectrum of the final desired product, PAMAM G4.5-piperazinyl-FITC dendrimer, presented an unexpectedly larger protons-integration number in the fluorescein region (6.0–8.0 ppm) (Fig. 2a). The synthesis procedure included extensive washing, of the derivatized dendrimer with water, and centrifugation with Microcon^®^ (cutoff of 10 kDa) in order to remove un-reacted FITC [33–37]. According to the structure of the intermediate PAMAM G4.5-*N*-Boc-piperazinyl, it was anticipated that only two units of FITC had been incorporated. However, the experimental ^1^H NMR data indicated, in all cases (n = 5), that near to 19 FITC molecules were added to PAMAM dendrimers with 17 of them probably occluded in non-covalent form.

**Fig. 3:**
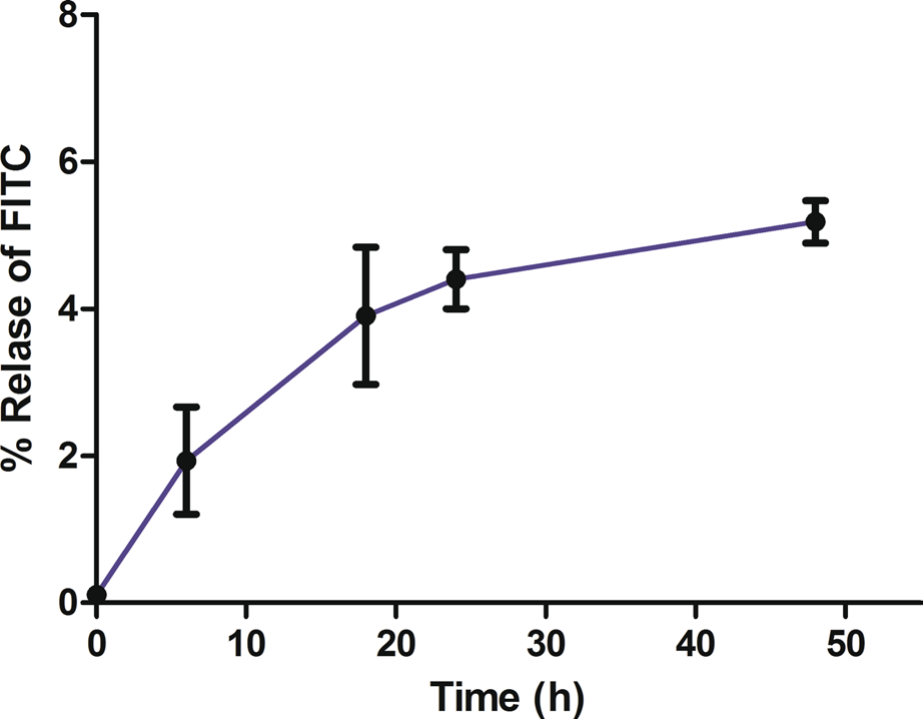
Release study of FITC from PAMAM G4.5-piperazinyl-FITC. The release of the encapsulated FITC study was made in PBS 1× at 37 °C, as described in “Methods” section, for the following periods of incubation: 0, 6, 18, 24 and 48 h. The fluorescence of the collected samples was measured in a plate reader with excitation and emission wavelength of 495 and 525 nm, respectively. Percentages of FITC released were calculated and* graphed*

In order to confirm this last observation, we also characterized the final products by MALDI-TOF mass spectrometry (Fig. 2b) observing a signal corresponding to the *m/z* of PAMAM G4.5 and to the *m/z* of PAMAM G4.5-piperazinyl-FITC dendrimer with up to 19 molecules of FITC. For these large molecules, the signals are not well resolved, but a general molecular weight distribution can be observed [38]. The FITC molecules could be covalently bound through the piperazine linker or occluded in the dendrimer. Because the synthetic dendrimer-piperazinyl intermediate presented a 1:2 stoichiometry (dendrimer:piperazine), only 2 FITC molecules could be bound covalently in the final product. The remaining FITC molecules would be non-covalently bound to the dendrimer. Figure 2b shows the MALDI-TOF experimental signal and the calculated signal for the dendrimer with 2, 14, 17 and 19 molecules of FITC. On the other hand, the FITC release from PAMAM G4.5-piperazinyl-FITC dendrimers was studied in aqueous solution. FITC encapsulated into dendrimers resulted to be very stable since the compound showed a low percentage of FITC release at 24 and 48 h (4.4 and 5.2 %, respectively). This means that this compound was stable at least for 48 h in this condition (Fig. 3).

The molecular encapsulation of FITC could be the result from hydrogen bonding interactions between the phenol, the isothiocianate, and the lactone moieties of this and the amines and amides functionalities of the polymeric amidoamine feature of PAMAM G4.5 [39]. No electrostatic bonding could be also considered as responsible, at least in part, of the encapsulation of fluorescent probe. In this case, one could speculate that the aromatic regions of FITC interact positively via van der Waals forces with the hydrocarbon backbone of PAMAM [40].

### PAMAM G4.5-piperazinyl-FITC dendrimer size

**Fig. 4:**
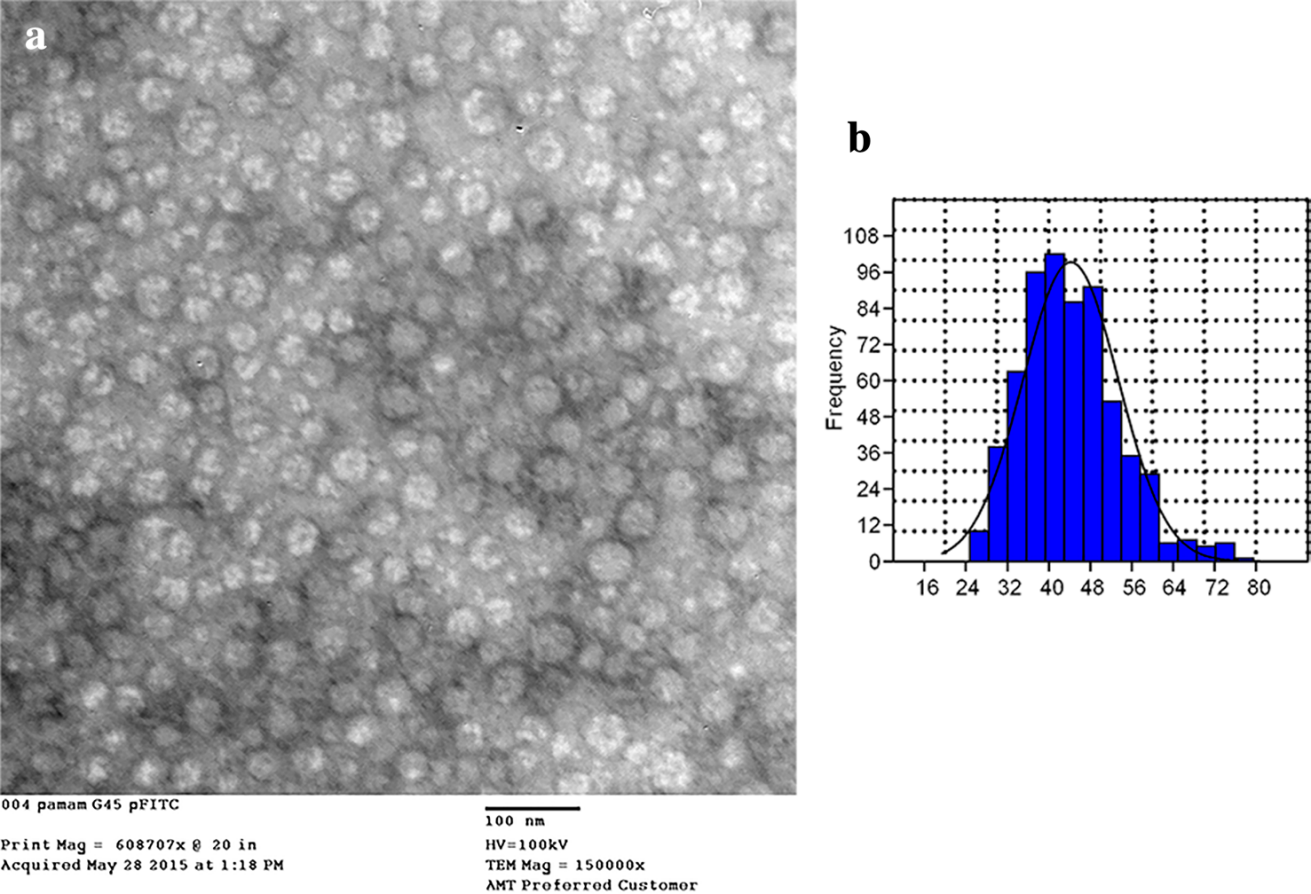
Size and morphology of PAMAM G4.5-piperazinyl-FITC. **a** Representative TEM image of nanoparticles stained with uranyl acetate. **b** Size distribution histogram from TEM images. Particle size was measured according to “Methods” section

The nanoparticle size distribution of PAMAM G4.5-piperazinyl-FITC dendrimer, was performed by DLS and TEM. PAMAM-FITC dendrimers hydrodynamic size was 96.3 ± 1.4 nm with a PdI of 0.0296 ± 0.0171. We also measured nanoparticle size by TEM, and could determine a mean PAMAM-FITC dendrimer diameter of 44.2 ± 9.2 nm (Fig. 4a, b). DLS provides the average hydrodynamic size of particles in a solution, being this measure directly related to the diffusive motion of the particles [41]. Thus, the size measured by DLS includes the solvent layers around the individual nanoparticles. These solvent layers are not present in TEM images of the nanoparticles due to previous sample drying [42]. Both techniques are complimentary and TEM gives information of nanoparticle structure as well. However, TEM suffered from the small sampling size involved [41]. The significant difference in size of PAMAM-FITC dendrimer measured by TEM and DLS, could be attributed to the presence of dendrimer solvent layers attached during DLS measurement. In addition, since the scattering intensity is directly proportional to the sixth power of the particle radius, DLS technique is extremely sensitive towards the presence of small aggregates [41]. Nevertheless, we do not think that dendrimer aggregation occurred due to the fact that polydispersion index obtained was close to zero.

### Cytotoxicity and hemolytic studies of PAMAM-FITC dendrimer

**Fig. 5:**
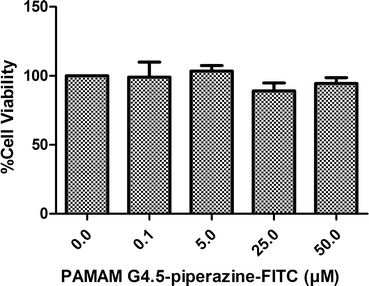
Cell viability study of PAMAM G4.5-piperazinyl-FITC on 4T1 cells. This study was carried out by MTT assay at different concentrations at 24 h of incubation as described in “Methods” section. Viability percentages and their error bars are expressed as mean ± SD and represent the result of three experiments. No cytotoxicity from FITC-conjugated PAMAM G4.5 dendrimers was observed on this cell line

The cytotoxicity of PAMAM G4.5-piperazinyl-FITC dendrimer was evaluated in 4T1 cells. These cells were incubated for 24 h with PAMAM- FITC dendrimer and no cytotoxicity was observed at the time period and concentrations assayed (Fig. 5).

Other authors who have evaluated the influence of surface modification in cytotoxicity of other type of PAMAM dendrimers, made these studies employing higher concentrations and different incubation times than the ones used in our work [19, 28].

PAMAM G4.5-piperazinyl-FITC dendrimers showed to be not cytotoxic up to 50 µM (maximal concentration tested). Anionic half generation dendrimers have previously been shown to be significantly less cytotoxic compared with cationic full generation dendrimers [43]. Kirkpatrick et al. [8], tested in vitro cytotoxicity of anionic half generation PAMAM dendrimers 3.5–6.5 and showed no cytotoxicity (IC_50_ > 100 μM). On the other hand, they also showed that 37 molecules of cisplatin could be bound to PAMAM G4.5 dendrimers [8]. Thus, we can speculate that if PAMAM G4.5-piperazinyl-FITC dendrimers could bind or encapsulate similar number of anti- tumor molecules per dendrimer, a dendrimer concentration of 50 µM would be sufficient enough to deliver drugs acting in the nM or few µM ranges.

**Fig. 6:**
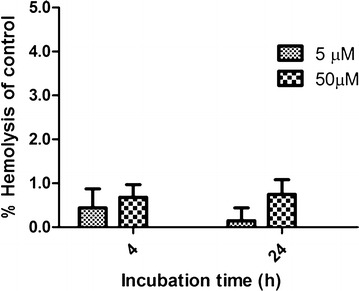
Hemolytic study of PAMAM G4.5-piperazinyl-FITC. The study was performed by incubating red blood cells from dog with 5 or 50 µM dendrimer concentrations at 4 and 24 h. The percent hemolysis was calculated considering the positive control of hemolysis (SDS 2 %) as 100 % of hemolysis

On the other hand, hemolytic studies showed that the incubation of red blood cells with PAMAM G4.5-piperazinyl-FITC dendrimers did not caused hemolysis. Thus, the compound showed to be neither cytotoxic nor hemolytic at 50 μM up to 24 h (Figs. 5, 6).

### In vitro cellular uptake of PAMAM G4.5-piperazinyl-FITC dendrimers

**Fig. 7:**
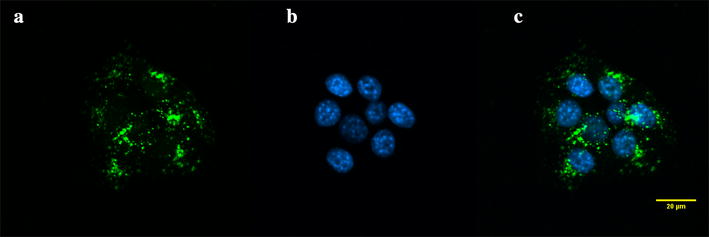
Cellular uptake of PAMAM G4.5-piperazinyl-FITC by 4T1 cells at 24 h incubation. **a** FITC stain (*green*), **b** DAPI counterstain (*sky blue*) and **c** merge. As it can be seen from the image, PAMAM G4.5-piperazinyl-FITC dendrimers were uptake by the cells. Magnification: 80×. *Scale bar* 20 μm

The cellular uptake of PAMAM G4.5-piperazinyl-FITC dendrimer was evaluated in 4T1 cells at 24 h of incubation. The dendrimers were uptaken by the cells (Fig. 7). Since another membrane selective dye was not used in the study, we cannot rule out the possibility that the dendrimer could be located also in the cell plasma membrane and in cell nuclei. However, most of the observed signal appears to be perinuclearly located (Fig. 7). Previously, our group had obtained experimental evidence that the distribution of PAMAM G4-FITC dendrimers in primary cultured human myometrial cells was perinuclear [15] and several reports have shown that intracellular distribution of different type of dendrimers was also perinuclear [44, 45].

Additionally, as previously mentioned, the result of the stability study of PAMAM G4.5-piperazinyl-FITC dendrimers showed that up to 24 h, no significant release of FITC was detected (Fig. 3). This result suggests that the fluorescence signal detected inside the cells by confocal microscopy is from the dendrimer linked FITC that were uptaken by the cells and not by free released FITC.

### Ex vivo fluorescence imaging of BALB/c mice bearing 4T1 tumor treated with PAMAM G4.5-piperazinyl-FITC dendrimer and tissue slide analysis

**Fig. 8:**
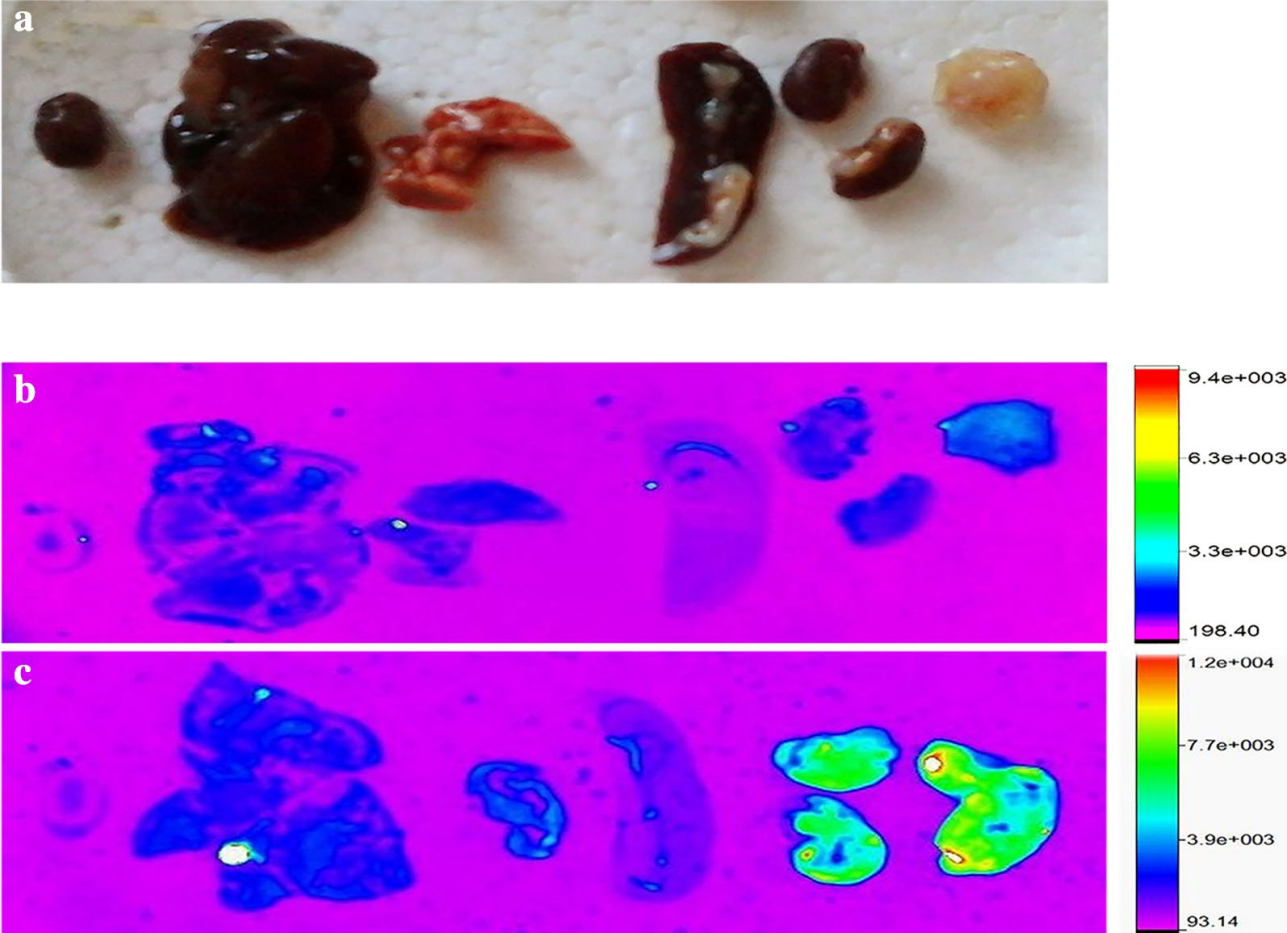
Ex vivo fluorescence imaging of BALB/c mice with 4T1 tumor and tumor tissue analysis. **a** Analyzed organs. The image was taken from organs of a control animal. **b** Ex vivo fluorescence image of organs from control animal. **c** Ex vivo fluorescence images of organs from PAMAM-FITC dendrimer-injected animals, 24 h post-injection. The images were taken at λ_exc_: 480 nm and λ_em_: 535 nm

**Fig. 9:**
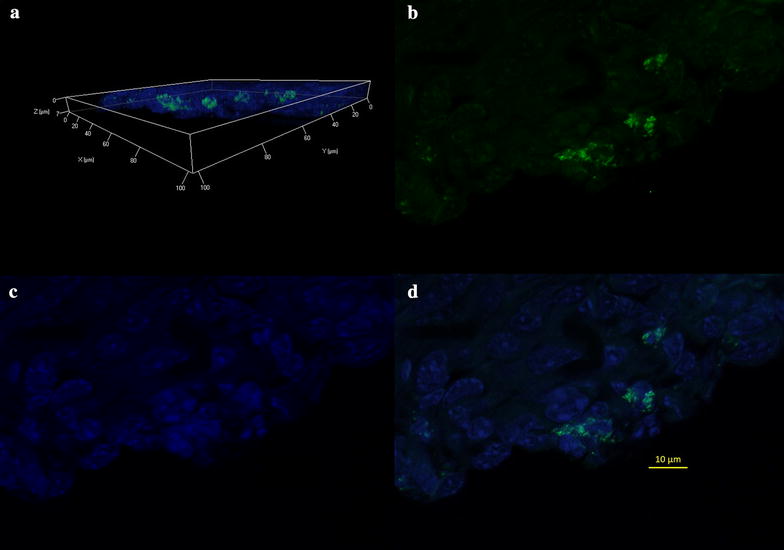
Tumor tissue imaging by LCM after 24 h PAMAM G4.5-piperazinyl-FITC dendrimer post-injection. **a** 3D reconstruction of tumor tissue section. PAMAM G4.5-piperazinyl-FITC dendrimers are shown in *green* and DAPI in* blue*. **b**, **c** and **d** 2D images of a slide from the 3D reconstruction of tissue section in part A, showing FITC stain, DAPI counterstain and merge, respectively. Magnification: 63×. *Scale bar* 10 µm

To the best of our knowledge, the present work is the first to investigate the uptake of PAMAM G4.5 dendrimers by mammary tumors. This study was made by ex vivo imaging through intravenous injection of PAMAM G4.5-piperazinyl-FITC dendrimer in BALB/c mice with 4T1 induced tumor. This tumor model resembles to that of human breast cancer stage IV, which is the metastatic stage [46]. Ex vivo imaging study was performed, because developed compound was labeled with FITC. FITC is a weak dye for in vivo study as the emitted fluorescence cannot cross the skin and hair of mice. However, the principal objectives of the present work could be achieved with the use of this dye. At 24 h post-injection, certain organs were excised and ex vivo images were taken. PAMAM G4.5-piperazinyl-FITC dendrimer showed a slightly hepatic uptake, renal excretion and tumor uptake (Fig. 8a–c). Renal depuration of this dendrimer is probably a result of its molecular weight (~34 kDa) which is below the threshold of renal filtration of 40–60 kDa. Tumor uptake may be due to enhanced permeability and retention effect (EPR effect), in which nanoparticles tend to accumulate more in tumor tissue than in the other tissues [7, 47–51]. We clearly observed that PAMAM G4.5 dendrimer was uptaken by tumor cells (Fig. 9). Thus, the developed compound labeled with FITC showed its capacity to be uptaken by the tumor. It could be interesting in the near future to label PAMAM G4.5-piperazinyl with Cy3 or Cy5 instead of FITC and try to perform in vivo imaging (bio-distribution) of the compound in this animal model.

## Conclusions

In order to evaluate half generation PAMAM dendrimers as possible vehicles in breast cancer, we developed and characterized PAMAM G4.5-piperazinyl-FITC dendrimers. These dendrimers were not cytotoxic in 4T1 tumor cells and were uptaken by these cells. Most importantly, when dendrimers were administered to BALB/c mice bearing 4T1 tumors, tumor uptake was observed. Hence, the results obtained by employing 4T1 tumor cells and mice with tumors induced with these cells, further suggest that the nanosystem enters tumor tissue cells.

One of the principal objectives of the present work was to verify that the developed nanosystem was able to enter tumor cells and be uptaken by the tumor. With the nanosystem PAMAM G4.5-piperazinyl-FITC dendrimer these objectives were accomplished.

### PAMAM G4.5 dendrimers are good candidates to be used as antitumor drug delivery systems for breast cancer treatment or diagnostics

Additionally, it is possible to employ PAMAM G4.5-*N*-Boc-piperazinyl dendrimers to generate other fluorescent imaging agents for in vivo studies of tumors. In this case, it would be necessary to bind these dendrimers with fluorophores that operate in the far-red and near-infrared region of electromagnetic spectrum (NIR fluorophores) [52]. The low absorption of light exhibited by tissues in this spectral window, makes it possible for the light to cross various centimeters [52]. The emitted FITC fluorescence cannot cross the skin and hair of mice, and therefore cannot be detected. The AAF fluorophore previously used to label PAMAM G4.5 dendrimers [32], which its excitation and emission wavelengths are 492 ± 3 and 514 ± 4 nm, respectively, exhibits the same limitations as FITC. Kim et al. 2013. conjugated the fluorophores Cy3 or Cy5 to PAMAM G5 and G6 dendrimers as imaging agents and as previously mentioned these could be a good option in imaging [53].

## Methods

### Synthesis of PAMAM G4.5-piperazinyl-FITC dendrimers

Methanol from PAMAM G4.5 dendrimers (Sigma Aldrich Co. 5 % w/v in methanol) was eliminated by vacuum evaporation (BUCHI). After that, the activation of the carboxyl groups of the dendrimers was made by EDC (1-ethyl-3-(3-dimethylaminopropyl)carbodiimide) addition (dissolved in PBS 1x) to previously dried dendrimers. The EDC:dendrimer ratio used was 30:1. This mixture was incubated for 30 min. *N*-Boc-piperazine dissolved in DMSO (30:1 *N*-Boc-piperazine:dendrimer ratio) was then added to the mixture and incubated for 3 h in the same conditions. The mixture was purified by gel filtration to eliminate unreacted *N*-Boc-piperazine and fractions of 500 μL were collected and controlled by thin layer chromatography (TLC). The fractions 5–9 were joined and lyophilized. According to ^1^H NMR analysis the rest of the fractions (1–4 and 10-end) did not correspond to the desired product. This procedure was performed five times and the results obtained were all the same.

The obtained intermediate, G4.5-piperazinyl-Boc, was dissolved in methanol. Dry dichloromethane was added to this solution followed by the addition of 1 µL of trifluoroacetic acid to eliminate Boc. The mixture was incubated in shaker for an hour. Solid sodium bicarbonate was added to the mixture, to eliminate trifluoroacetic acid from the solution, and shake for 15 min and the supernatant transferred to another vial. Solid sodium bicarbonate was washed twice by adding 100 μL of methanol each time, joining these supernatants to the first one which was transferred to a vial. The organic solvent was eliminated by vacuum evaporation and 250 µL of PBS 1× and 1 mg of FITC (dissolved in DMSO) was added and incubated overnight. The mixture was purified by gel filtration and fractions of 500 μL were collected and controlled by TLC. The fractions 5–9 were joined and non-covalently bound FITC to the dendrimers was eliminated by three cycles of washing with distilled water and centrifugation with a centrifugal filter (cutoff of 10 KDa, Microcon^®^) (at 12,000 rpm by 10 min) [33–37]. Finally, the obtained supernatant was lyophilized. According to ^1^H NMR analysis the rest of the fractions (1–4 and 10-end) did not correspond to the desired product. This procedure was performed five times and the results obtained were all the same.

## ^1^H NMR spectroscopy

The H^1^ NMR spectrums were recorded, at 303 K, in Bruker DPX-400 (400 MHz) spectrometer, employing deuterium oxide (D_2_O) as solvent. The number of scans used to obtain the spectrums was of 64. The chemical proton shifts are expressed in ppm (δ) employing TSM (tetramethylsilane) as zero.

### MALDI-TOF mass spectrometry

Mass spectrometry was performed on a MALDI-time of flight mass spectrometer (Microflex LR, Bruker Daltonik Billerica, MS, USA) equipped with a 337 nm Nitrogen laser, operated in positive-ion, reflectron mode. External mass scale calibration was performed with Protein Calibration Standard Mix (Bruker). Acquisition was performed with FlexControl software (version 3.4, Bruker Daltonik GmbH).

The matrix used in the experiments was 2,5-dihydroxybenzoic acid (DHB) with D-(þ)-fucose (1:1).

### Dynamic light scattering

The particle size, size distribution and particle charge of PAMAM G4.5-piperazinyl-FITC dendrimer was measured by DLS (Zetasizer Nano ZS, Malvern Instruments, UK). Briefly, PAMAM G4.5-piperazinyl-FITC dendrimer was dissolved with deionized water and the sample was filtered with a 0.22 μm membrane. The data obtained were the results of three experiments of 5 measures each. Data are expressed as mean ± SD.

### Transmission electron microscopy

An aliquot of PAMAM G4.5-piperazinyl-FITC dendrimer was dropped on a carbon coated-copper grid, after 1 min the excess of liquid was removed and the grids were stained with uranyl acetate. Imaging was performed immediately after air-drying by TEM (JEOL JEM-1010). Nanoparticle size measurement was performed by Image J and the quantification by PAST version 2,12. The data obtained were the results of three experiments.

### Release study of FITC from PAMAM G4.5-piperazinyl-FITC dendrimer

The release study of FITC was made by using a Slide-A-Lyzer Mini Dialysis unit Plus Float of 10 kDa MWCO (Thermo Scientific). Briefly, 100 μL of 2.5 mg/mL of PAMAM G4.5-piperazinyl-FITC dendrimer solution was introduced in the dialysis unit (inner phase) which was immersed in 10 mL of PBS 1× pH = 7.4 (outer phase). The FITC release study was made at 37 °C with continuous agitation of the outer phase. Samples of 100 μL were taken from the outer phase at the following periods of time: 0, 6, 18, 24 and 48 h. After each sampling, the outer phase was replenished with 100 μL of PBS 1× pH = 7.4. The fluorescence of the initial solution of dendrimers and of the samples collected was measured in a plate reader with excitation and emission wavelengths of 495 and 525 nm, respectively. Percentages of FITC released were calculated and graphed. The data obtained were the results of three experiments and are expressed as mean ± SD.

### Cytotoxicity

The potential PAMAM G4.5-piperazinyl-FITC dendrimer cytotoxicity was evaluated in mouse mammary tumor cells, 4T1 (ATCC). This study was carried out by MTT assay of cell viability, according to the method described by Mosmann [54]. Briefly, 5 × 10^3^ 4T1 cells were seeded in a 96 well plaque and after 24 h the cells were treated with different concentrations of PAMAM G4.5-piperazinyl-FITC dendrimer. The absorbance was measured in a plaque reader at 570 nm. The cell viability percentages of different treatments were calculated by considering the absorbance of control as 100 % viability. Presented results correspond to n = 3 performed in triplicate and expressed as mean ± SD. There is no significant difference between cells treated with different concentrations of PAMAM G4.5-piperazinyl-FITC dendrimer and control cells at 24 h (Student′s t test: P < 0.05).

### Hemolysis

Hemolysis of PAMAM G4.5-piperazinyl-FITC dendrimer was assayed according to Prieto et al. [55]. Briefly, freshly prepared blood from a healthy dog was centrifuged at 2000*g* for 15 min at 4 °C to separate red blood cells from plasma. The plasma was replaced with the same volume of PBS 1× pH = 7.4. 200 µL of red blood cells were incubated with 5 µM or 50 µM of PAMAM G4.5-piperazinyl-FITC dendrimer for 4 or 24 h at 37 °C. After the incubation, samples were centrifuged at 2000*g* for 10 min and the supernatant absorbance was measured at 414 nm with NanoDrop1000 (Thermo). Hemolysis was expressed as a percentage of the hemoglobin release induced by SDS 2 %. The data obtained were the results of three experiments and are expressed as mean ± SD.

### Cell uptake

For uptake studies, 5 × 10^3^ 4T1 cells were growth on coverslips for 24 h. After this period, cells were treated with 10 μg/mL of PAMAM G4.5-piperazinyl-FITC dendrimer and incubated for 24 h at 37 °C and 5 % CO_2_. Cells were washed with PBS 1× and fixed for 15 min with PFA 3 % (paraformaldehyde). The cells were counterstained with DAPI (1 µg/mL). Cells were mounted (ProLong Antifade Kit (P7481) of Molecular Probes-Invitrogen) over slides and observed by LCM (laser confocal microscopy) at the following wavelengths: 488 and 405 nm.

### Ex vivo fluorescence imaging of tumor

The experimental procedures with BALB/c mice, were approved by the local ethics committee (CHEA, Uruguay, protocol number: 071140-000207-12), in accordance with national legislation. A tumor uptake study of PAMAM G4.5-piperazinyl-dendrimers was made by the intravenous injection of 15 mg/kg of the nanosystem in BALB/c mice with 4T1 tumors. At 24 h post-injection, we went on to extract the following organs: heart, liver, spleen, lungs, kidneys and tumor. The organs were disposed in a petri dish and then inside the fluorescent imaging equipment (In vivo MSF X PRO, Bruker). The fluorescence of the before mentioned organs was captured with and excitation and emission wavelength of 480 and 535 nm, respectively.

### Tumor section analysis by LCM

After 24 h post-injection of PAMAM G4.5-piperazinyl-FITC dendrimers in mice, the tumor was fixed with PFA 3 % for 1 h, thoroughly washed and cryoprotected with 15 and 30 % sacarose for 1 h, respectively. The sample was introduced in freezing medium and stored at −20 °C. Tissue sections of 5 µm were obtained with a cryostat (Slee Mainz 2 Cryostat Mev), counterstained with DAPI and analyzed by LCM by using 405 and 488 nm lasers, respectively.

## Abbreviations

AAF: 5-(aminoacetamido)fluorescein
DAPI: 4′,6-diamidino-2-phenylindole
DLS: dynamic light scattering
EDC: 1-ethyl-3-(3-dimethylaminopropyl) carbodiimide
FITC: fluorescein isothiocyanate
PAMAM-FITC dendrimers: PAMAM G4.5-piperazinyl-FITC dendrimers
^1^H NMR: proton nuclear magnetic resonance
LCM: laser confocal microscopy
MALDI-TOF mass spectrometry: matrix-assisted laser desorption/ionization- time-of-flight mass spectrometry
PAMAM: poliamidoamine
PdI: polydispersion index
TEM: transmission electron microscopy
TFA: trifluoroacetic acid
TLC: thin layer chromatography
TSM: tetramethylsilane
